# Framing a pig welfare assessment protocol suitable for smallholder settings in low-to-middle income countries

**DOI:** 10.1007/s11250-026-05131-5

**Published:** 2026-06-12

**Authors:** Lauren Oehlers, Rebecca Doyle, Phil C. Glatz, Jenny-Ann Toribio

**Affiliations:** 1 Sydney School of Veterinary Science, Faculty of Science, The University of Sydney, Sydney, NSW 2006 Australia; 2 The Royal (Dick) School of Veterinary Studies, The University of Edinburgh, Edinburgh, Scotland; 3 International Livestock Research Institute, Addis Ababa, Ethiopia; 4 29 Russell Avenue, Hazelwood Park, SA 5066 Australia

**Keywords:** Animal welfare, Research for development, Smallholder pig production

## Abstract

Smallholder pig production in low-to-middle income countries (LMIC) can be an important contributor to the livelihoods of families including income, food, social and cultural obligations. However, to date there has been scant evaluation of welfare in smallholder pig production systems in these countries. To contribute to research to address this knowledge gap, we conducted a review of existing pig welfare assessment protocols to determine if any existing protocol is directly applied to smallholder pig production systems in LMIC. A total of 16 protocols were identified and evaluated but none were suited to the smallholder context, because they were created for commercial, conventional intensive pig production systems in developed countries. Therefore, a preliminary version of a new protocol was formulated drawing upon the indicators in the existing frameworks relevant to smallholder systems. The new protocol consists of a questionnaire (15 questions) and an observation record sheet (23 welfare indicators). Of the welfare indicators, 5 are designated as critical indicators in urgent need of immediate intervention to improve pig welfare when ≥ 1 of these measures has a zero score. The critical indicators are: 1) Access to water, 2) Access to feed, 3) Body condition score, 4) Space allowance, 5) Negative social behaviour. We propose that the preliminary protocol be piloted in various smallholder pig contexts to refine and validate it. The new protocol is an essential first step toward better understanding of the welfare of the pigs in smallholder pig production systems in LMIC.

**Supplementary information:**

The online version contains supplementary material available at 10.1007/s11250-026-05131-5.

## Introduction

Animal welfare is a multidimensional concept embracing freedom from suffering, the ability to exhibit a high level of biological functioning and positive experiences (Alonso et al. 2020; Maes et al. 2020). Concerns about animal welfare are at the forefront in commercial livestock industries worldwide, and are an important factor influencing animal management, especially in intensive production systems (Cronin et al. 2014; Lawrence et al. 2024). This is in part due to societal pressure on livestock producers to meet the welfare standards in Codes of Practice along with recognition of production benefits associated with ensuring adequate welfare for their animals (Alonso et al. 2020; Cornish et al. 2020).

A world recognized conceptual model of animal welfare is the Five Domains Model (FDM). Today this model, created in 1994 and updated with advances in welfare science, incorporates five domains in which welfare can be either compromised or enhanced (Mellor et al. 2020). These domains include three centered on internal survival-related factors (nutrition, physical environment, health), one on external situation-related factors (behavioural interactions), and a fifth domain that considers how the four physical domains impact the affective experience of the animal (mental state) (Table 1). Importantly, this model makes clear that minimizing negative physical or mental states does not necessarily result in positive welfare for an animal (Mellor and Beausoleil 2015).

**Table 1:** Five domains model for animal welfare assessment with key features and examples as presented in Mellor and Beausoleil (2015) and Mellor et al. (2020)

Physical/Functional Domains							
Survival-related factors						Situation-related factors	
1. Nutrition		2. Physical Environment		3. Health		4. Behavioural interactions	
Restrictions on:	Opportunities to:	Unavoidable/Imposed conditions:	Available conditions:	Presence of:	Little or no:	Exercise of voluntary actions impeded by:	Voluntary actions exercised by:
Water or food intake	Drink enough water	Thermal extremes	Thermally tolerable	Disease	Disease	Choices markedly restricted	Available engaging choices
Food variety and quality	Eat enough food	Unsuitable substrate	Suitable substrate	Injury	Injury	Constraints on animal-animal interactive activity	Bonding/play
		Close confinement	Space for freer movement	Functional impairment	Functional impairment	Limits on threat avoidance, escape or defensive activity	Using refuges, retreat, or defensive attack
Voluntary overeating	Eat a balanced and varied diet	Environmental monotony	Normal environment variation	Obesity/leanness	Appropriate body condition score	Limitations on sleep/rest	Sleep/rest sufficient
		Unpredictable events	Predictability	Poor physical fitness	Good fitness level	Constraints on environment focused activity	Free movement/exploration/foraging/hunting
						Human-animal interaction	Positive human-animal interaction
**Affective Experience Domain**							
**5. Mental state**							
Negative:	Positive:	Negative:	Positive:	Negative:	Positive:	Negative:	Positive:
		Forms of discomfort:	Forms of comfort:			Anger, frustration	Calmness
Thirst	Quenching thirst	Thermal	Thermal	Pain	Comforts of good health and high functional capacity	Boredom, helplessness	Engaged, in control
Hunger	Pleasure of different tastes/smells	Respiratory e.g., breathlessness	Respiratory	Debility		Loneliness, isolation	Maternally rewarded
		Auditory e.g., impairment	Auditory	Sickness		Depression	Playfulness
		Visual e.g., glare/darkness eye strain	Visual	Physical exhaustion	Vitality of fitness	Exhaustion	Energized/refreshed
						Uncertain/fearful interaction with humans	Confident/skillful interaction with humans

Pig production is the second most important livestock sector globally, after poultry meat production (FAO 2024). As 34% of all meat produced worldwide is pork (FAO 2024), there is consumer demand to increase production which has led to intensive commercial farms characterised by high biological and economic productivity with simultaneously low input of labour, feed and space per animal (Maes et al. 2020). In countries where animal welfare is included in legislation and Codes of Practice exist, pig producers and the pig industry more widely need to consider pig welfare in routine management and slaughter practices.

In low-to-middle income countries (LMIC), a rise in consumer demand for pork, particularly evident with improvement in the socioeconomic status of households in urban centres (Clonan et al. 2016), is seeing a shift toward intensive commercial piggeries. Accompanying this, there is growing consumer awareness of animal welfare in some countries in South America, China and Mexico (Carnovale et al. 2021; Estévez-Moreno et al. 2021, 2022). But in the majority of LMIC countries animal welfare legislation is poor or non-existent (World Animal Protection 2020) and action is needed to raise awareness of consumers and farmers along with introduction of regulatory and legislative controls. Among these countries, even those with high pork consumption, the smallholder pig sector contributes substantially to pork production providing, for example, 76.2% of pigs raised in the Philippines in 2022 (Philippine Statistics Authority 2023a). At present and for the foreseeable future, the smallholder pig sector is expected to remain in existence and to continue to be important for household income security and sociocultural purposes in numerous countries across Africa, Asia and the Pacific (Amben et al. 2017; Bettencourt et al. 2015; Ouma et al. 2017; Sharifuzzaman et al. 2024). Yet little to no consideration has been given to pig welfare in these smallholder contexts which range from households with scavenging pigs to small-scale penned pig herds (Food and Agriculture Organization of the United Nations, World Organisation for Animal Health, World Bank 2010).

Examples of smallholder pigs in LMIC include free-roaming indigenous pigs that scavenge for food reared by rural households for subsistence and cultural purposes, crossbred pigs confined by tethering or in simple pens raised for consumption and local sale by rural and urban households, and fully confined exotic and crossbred pigs raised as a primary source of household income. The variation in management and size of smallholder pig herds results in different definitions for a smallholder pig herd between countries, for example, up to 2 sows in Vietnam (General Statistics Office of Vietnam 2020), up to 10 sows in the Philippines (Philippine Statistics Authority 2023b), and less than 50 pigs total in Thailand (Woonwong et al. 2020).

In smallholder systems, inadequate feeding, lack of veterinary care and poor breeding management are some of the major impediments to pigs expressing their full performance potential (Dione et al. 2014; Huynh et al. 2006; Mbuthia et al. 2015). Animal health problems also contribute to significant economic losses, which can be in the form of high mortality, poor animal performance and lost market opportunities (Cronin et al. 2014; Kimbi et al. 2015; Leslie et al. 2015). These constraints on pig health and production indicate detrimental impacts on the welfare of smallholder pigs in some settings. This, along with the large number of pigs kept by smallholders, provides real impetus for research on smallholder pig welfare. In recent years, the need for tools to evaluate welfare in smallholder livestock systems has been voiced, in recognition that benchmarking animal health, production and welfare in smallholder systems, is the essential foundation for co-design with communities of context appropriate interventions (Doyle et al. 2021).

In view of this need for research on pig welfare in smallholder pig systems, the objectives of the work presented in this paper were 1) to review existing assessment protocols used to evaluate pig welfare in pig production systems to determine whether existing protocols are applicable to smallholder pig settings, and, if not fit for purpose, 2) to develop a preliminary version of a pig welfare assessment protocol specific for smallholder pig systems in LMIC.

## Materials and methods

### Review of existing welfare assessment protocols

A review was conducted using the following structured methodology to identify existing pig welfare assessment protocols and to consider their respective suitability for use on smallholder pig farms in LMICs.

In accordance with the PRISMA guidelines for systematic reviews (Liberati et al. 2009), to identify existing pig welfare assessment protocols four databases (Commonwealth Agricultural Bureaux (CAB) Abstract, Scopus, Medline, Global Health) were searched for a combination of pig, welfare and assessment protocol and their respective synonyms joined by Boolean operators, with no restriction on year of publication (Table 2). The four databases were selected to ensure coverage of publications in the humanities and sciences, specifically in veterinary medicine. Inclusion criteria consisted of any publication that described use of a welfare assessment protocol in pig production systems on farm (Table 3).

**Table 2:** Search strategy and search terms used for examining scientific databases for a review conducted in 2021 of the literature to identify existing pig welfare assessment protocols

Databases searched	Search terms
CAB Abstracts via Web of Science (1910 – present) 1145 results	(pig OR pigs OR swine OR sow OR sows OR boar OR boars) AND (welfare OR well-being OR “quality of life”) AND (assessment OR protocol OR guidelines OR code OR framework), mapped to topic
Scopus 272 results	(pig OR pigs OR swine OR sow OR sows OR boar OR boars) AND (welfare OR well-being OR “quality of life”) W/3 (assessment OR protocol OR guidelines OR code OR framework), mapped to article title, abstract and keywords
Medline via Ovid (1964 – present) 374 results	(pig OR pigs OR swine OR sow OR sows OR boar OR boars) AND (welfare OR well-being OR “quality of life”) AND (assessment OR protocol OR guidelines OR code OR framework), mapped to key words and subject headings
Global Health via OvidSP (1910 – present) 83 results	(pig OR pigs OR swine OR sow OR sows OR boar OR boars) AND (welfare OR well-being OR “quality of life”) AND (assessment OR protocol OR guidelines OR code OR framework), mapped to key words and subject headings

**Table 3:** Inclusion and exclusion criteria used to evaluate the 1385 records returned from online database and grey literature search in 2021 for existing pig welfare assessment protocols

Inclusion	Any publication that describes and implements or describes the approach to implementation of a welfare assessment protocol for use in pig production systems on farm.
Exclusion	Publication not relating to swine, insufficient detail included to allow evaluation of application to assess welfare in pig production systems on farm, not available in English, and full text not available.

The grey literature search plan involved consultation with an animal welfare expert to identify sources important to search for relevant literature that met the inclusion criteria for this project (Table 3). The sources searched and literature identified are outlined in Table 4.

**Table 4:** Grey literature search sources identified through consultation with an animal welfare expert and the reason for their inclusion among the existing pig welfare assessment protocols identified in 2021

Source searched	Literature included	Reason for inclusion
Council of European Union (EU)	Council Directive 2008/120/EC of 18 December 2008 laying down minimum standards for the protection of pigs	Important legislation for the European Union that is enforced by law. The Directive lays down minimum standards for the welfare of pigs. The competent authority of each Member State is to conduct inspections on a regular basis to check that the provisions of this Directive are being complied with.
Department for Environment, Food and Rural Affairs (DEFRA)	Code of Practice for the Welfare of Pigs	Adherence to the Code recommendations will help keepers to maintain the standards required to comply with animal welfare legislation in England. During on-farm welfare inspections carried out by the Animal and Plant Health Agency and Local Authorities, inspectors assess compliance against legislation and this Code.
World Organisation for Animal Health (OIE)	Terrestrial Animal Health Code - Animal Welfare and Pig Production Systems	The OIE Terrestrial Animal Health Code provides standards for the improvement of animal health, animal welfare and veterinary public health to be applied by OIE Member Countries to safeguard the health and welfare of terrestrial animals and ensure the safety of international trade in animals and animal products. Chapter 7.13 of the Code is an important publication internationally addressing the welfare aspects of commercial domestic pig production systems.

The welfare assessment protocol described in each publication meeting the inclusion criteria was then individually evaluated for suitability for use on farm in smallholder pig settings. The evaluation consisted of three steps.

1. Comparison against the FDM (deemed the gold standard by the author team) to ascertain whether the protocol was comprehensive (that is, including indicators of the 4 physical domains) and robust (that is, including indicators that can be recorded on farm in a consistent and reliable manner).
2. Previous application in extensive pig management system/s in any country (Yes/No) as a marker that all/some of its component welfare indicators could be measured for pigs kept outdoors.
3. Feasible for implementation on smallholder pig farms in LMIC (Yes/No), including consideration of the low resource context (such as no/limited access to specialized mobile equipment or a diagnostic laboratory) and diversity of pig management (free-roaming, extensive confinement, semi-intensive confined).

### Development of new assessment protocol

Based on the review findings, a preliminary version of a new on-farm welfare assessment protocol was developed by extracting, refining and collating the indicators from the existing reviewed protocols that were context appropriate for a LMIC smallholder pig farm, and in combination would provide a reliable assessment of pig welfare across the four physical domains of the FDM.

For design of the preliminary assessment protocol, a smallholder pig farm in a LMIC was characterised by an average herd size of five pigs (sows, piglets, growers, finishers) including 0–2 sows, often with suckling piglets making up the largest proportion of total herd size (Kimbi et al. 2015; Leslie et al. 2015; Samkol et al. 2006; Smith et al. 2019).

## Results

### Review of existing welfare frameworks

**Fig. 1:**
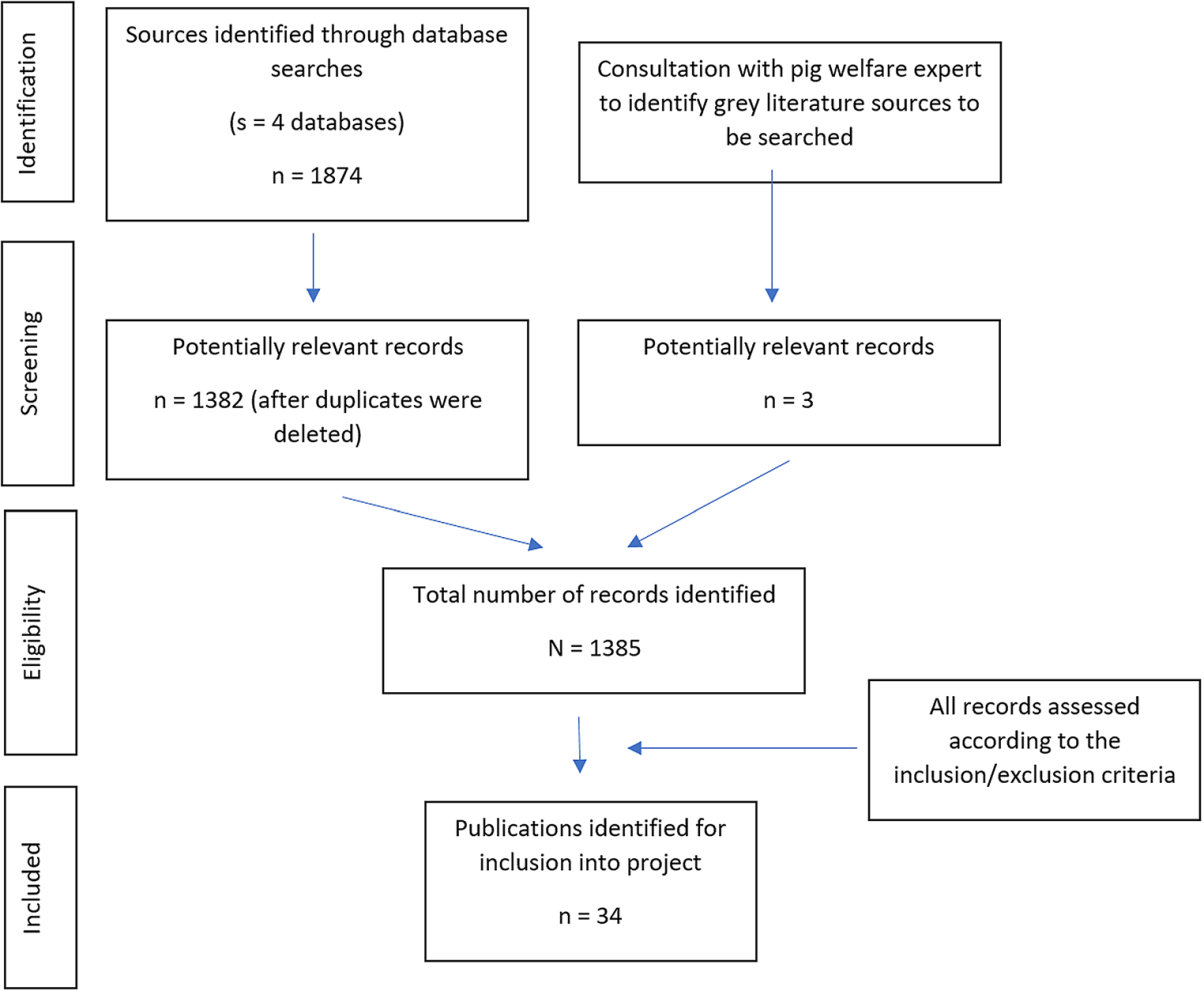
Flow diagram of the review of literature performed to identify, screen and assess records for eligibility for inclusion in a review of existing pig welfare assessment protocols

The database and grey literature searches identified an initial set of 1874 and 3 records respectively (Fig. 1) with 34 eligible publications (details of each provided in Supplementary information 1). Across these, it was determined that 16 assessment protocols were described for use to assess one or more pig production systems in a total of 19 countries, with the most commonly applied being the Welfare Quality® protocol (18 publications) (Table 5).

**Table 5:** List of the 16 welfare assessment protocols described in the 34 eligible publications and the findings from the three-step evaluation of each protocol

Welfare assessment protocol	Number of publications describing application of the protocol	List of countries where protocol applied (number of publications by country)	Summary of protocol comparison to the Five Domains Model to ascertain whether comprehensive and robust^a^	Application in extensive pig management system^b^ (Y/N)	Feasible for implementation on smallholder pig farms in low-to-middle income countries (Y/N)
WQ® protocol	18 (Alpigiani et al. 2016; Andronie et al. 2014; Czycholl et al. 2017, 2016a, 2016b, 2017, 2018; Dippel et al. 2014; Friedrich et al. 2019a, 2019b; Losada-Espinosa et al. 2017; Martín et al. 2017; Munsterhjelm et al. 2015; Scott et al. 2009; Temple et al. 2012, 2011, 2013; Van Staaveren et al. 2018)	Austria (1) Denmark (1) Finland (1) France (2) Germany (9) Ireland (1) Italy (1) Mexico (1) Netherlands (1) Romania (1) Spain (3) Sweden (1) United Kingdom (2)	The WQ protocol which is comprised mainly of animal-based indicators, when implemented on farm in full provides comprehensive coverage of Domains 1–4 with consistent, reliable data on the majority of indicators when implemented by trained evaluators. Indicators shown to have unsatisfactory reliability in > 1 publication were: •Individual animal indicators - bursitis, skin condition •Qualitative Behaviour Assessment (QBA) •Human-animal relationship (HAR) test.	Yes Finishing pigs – semi-free range (Alpigiani et al. 2016)	No Due to Long duration of time required for the welfare assessment (6 hours for full implementation) •Need for observer proximity to pigs for close, uninterrupted observation •Need for farm records (eg mortality) •The complexity of the QBA scoring 20 descriptors using a visual analogue scale •Considerable training required for evaluators.
Animal Welfare Indicators: Practical Guide—Pigs Kuratorium für Technik und Bauwesen in der Landwirtschaft e.V. (KTBL)	2 (Friedrich et al. 2020a; Pfeifer et al. 2020)	Germany (2)	Comprehensive coverage of Domains 1–4 for sows but is limited to Domains 1–3 for piglets and fattening pigs so no information on animal behaviour for these pigs. Among the indicators assessed there is a higher proportion of management-based indicators plus use of litter-level measures for piglets rather than animal-level. Indicators considered to have acceptable reliability.	No	No Although the lower number of indicators means KTBL is more feasible than the WQ® protocol, the inclusion of management-based indicators means that farm records are required.
Iceberg indicators protocol	1 (Friedrich et al. 2020b)	Germany	Proposes a small set of ‘iceberg’ indicators for welfare assessment of sows and of piglets based on Hierarchical Component Model analysis of farm data for the WQ® protocol and the KTLB protocol. Not comprehensive coverage as proposed set of iceberg indicators for sows assesses only Domains 2–4 and for piglets assesses only Domains 1&3. Evaluation of reliability is stated to be required in future work.	No	No Although a simplified assessment with a low number of indicators enhances the feasibility in terms of time requirement and complexity, the inclusion of management-based indicators means farm records are required.
Animal Needs Index (ANI)	1 (Annen et al. 2011)	Austria, Germany	ANI protocol to conduct assessment of fattening pigs is reasonably comprehensive consisting of 5 components that assess Domains 2–4 and indirectly Domain 1, with a focus on environmental and management-based indicators. No comment on consistency or reliability of indicators.	Yes Fattening pigs – free range (Annen et al. 2011)	No Although it is a simplified assessment tool with consideration for extensive management systems, it includes multiple indicators that require data from farm records.
Farm Welfare Index (FWI)	1 (Barbari et al. 2008)	Italy	FWI assesses 3 categories: general data, buildings, and pig categories (mating/pregnancy, farrowing/lactation, weaning, fattening/breeding). Comprehensive coverage of Domains 1–4 for sows and fattening pigs. No comment on consistency or reliability of indicators.	No	No Although it is a simplified assessment tool based predominantly on inspector observation, it requires a 2–4 hour farm visit and use of the FWI software.
Guidelines for Swine Keeping	1 (Ben-Dov et al. 2014)	Israel	Consists of 22 guidelines with emphasis on meeting stated requirements for building/facility, staff, equipment, feed and water. Reasonably comprehensive coverage of Domains 1–3 but minimal for Domain 4. No comment on consistency or reliability of guidelines but given present/absent nature of guidelines, there is little ambiguity.	No	No Although assessment involves a relatively short on-farm visit, the guidelines are heavily based on intensive indoors management system.
German National Assessment Catalogue for Animal Husbandry (NACAH)	1 (Bergschmidt and Schrader 2009)	Germany	Consists of 24 behavioural indicators grouped in functional systems: Social behaviour, Locomotion, Rest and sleep, Feeding, Elimination, Comfort behaviour, Exploration. Not comprehensive coverage as assesses only Domains 1,2,4 for fattening pigs. No comment on consistency or reliability of indicators but assessment is based on farmer report of housing.	Yes Extensive - fattening pigs (Bergschmidt and Schrader 2009)	No Although NACAH can be implemented based on farmer report without a farm visit, farm classification for pig behaviour is based on the commercial pig housing systems present in Germany.
Sow Welfare (SOWEL)	1 (Bracke et al. 2002)	Netherlands	Data on 37 attributes of farm housing and management are entered to SOWEL software to obtain a welfare score. Comprehensive assessment of Domains 1–4 based on housing and management. No comment on consistency or reliability.	Yes Outdoor huts - Pregnant sows	No Although assessment involves only on-farm observation and farmer report of housing and management. It requires use of SOWEL software and levels per attribute are heavily based on commercial pig housing systems in The Netherlands.
Bien-Être en Élevage de Porcs (BEEP) – translates to welfare in pig farming	1 (Courboulay et al. 2020)	France	Consists of 12 indicators assessed by the farmer, some based on pig group assessment and others on individual pig assessment. Comprehensive assessment of Domains 1–4. No evaluation of tool consistency or reliability.	No	No Although an easy-to-use tool for commercial farmers to implement after short training, it is designed for confined commercial pig housing and management systems.
‘Real Welfare’ Scheme	1 (Pandolfi et al. 2017)	United Kingdom	To assess pig welfare on finishing pig farms, RW consists of 5 animal-based indicators assessed at pen-level - Pigs requiring hospitalization, Lame pigs, Pigs with tail lesions, Pigs with body marks, Enrichment use. Not comprehensive as indicators cover only Domains 3–4. Good inter-observer reliability assumed but not measured.	Yes Outdoor (shelter+field) – Finishing pigs (Pandolfi et al. 2017)	No Although it involves observation of pig groups during a short on-farm visit, indicators are based on observation of multiple pig groups based on standard United Kingdom (UK) pig farms and there is no consideration of Domains 1–2 or relevant resource- or management-based indicators.
Herd Health and Welfare Index (HHWI)	1 (Wadepohl et al. 2019)	Germany Netherlands Belgium Bulgaria Denmark France Italy Poland Switzerland	Consists of 5 animal-based indicators (bursa alterations, lameness, manure on body, runts, tail/ear/flank biting) for weaner pigs and for fattening pigs to ascertain the overall HHWI points per farm. Not comprehensive coverage as limited to Domains 1&3 and for two categories of pigs on farm. Demonstrated reasonable inter-observer reliability within country.	No	No Although a simplified assessment with reduced indicators and completed on-farm in a 2-hour visit by a trained assessor, the index is designed for intensive indoors management system.
Model Code of Practice for the Welfare of Animals Pigs Third Edition PISC Report 92 Australia	1 (Primary Industries Standing Committee 2008)	Australia	The Code designates Standards for food and water, accommodation, equipment, environment, protection, waste control, pigs kept outdoors, inspections, health, farrowing and weaning, moving pigs, elective husbandry procedures, preparation for transport and slaughter, emergency euthanasia. Comprehensive coverage of Domains 1–4.	Yes Extensive outdoors – all pig categories	No As use of the Standards for welfare assessment involves an on-farm visit of long duration and extensive discussion with farmer. Plus the Standards are written based on commercial pig housing and management systems present in Australia.
Scientific opinion on the use of animal-based indicators to assess welfare in pigs	1 (EFSA Panel on Animal Health and Welfare 2012)	European Union	Provides a short list of indicators for the initial welfare assessment for 3 pig categories. The respective short list is: Comprehensive for sows and boars covering Domains 1–4. Not comprehensive for fattening pigs (missing Domain 1) or for piglets (missing Domain 4).	No	No Although using a short list of indicators per pig category for the on-farm visit and discussion with farmer, the evaluation of indicators is based on commercial pig farming and housing systems present in countries of the European Union.
Council Directive 2008/120/EC – minimum standards for the protection of pigs	1 (Council of the European Union 2009)	European Union	The Directive is comprehensive covering Domains 1–4 with particular emphasis on Domain 2 housing design impacts on welfare and health of the animals.	No	No A welfare assessment based on the Directive involves an on-farm visit of long duration and extensive interview with farmer. Plus the Directive Articles are written based on intensive pig housing systems present in countries of the European Union
Code of Practice for the Welfare of Pigs	1 (DEFRA 2020)	England	The Standards are comprehensive covering Domains 1–4 with detailed sections on Stockmanship & staffing, Health & welfare, Disease control & biosecurity, Emergencies, Inspection, Handling, Tethering, Transport, Marking, Responsible medicines usage & record keeping, Accommodation, Management, Mutilations, Additional specific recommendations per pig category, Outdoor husbandry systems.	Yes Extensive outdoor – all pig categories	No A welfare assessment based on the Standards involves an on-farm visit of long duration and extensive interview with farmer. Plus the Standards are written based on commercial pig housing and management systems present in England.
Terrestrial Animal Health Code – Animal Welfare and Pig Production Systems	1 (OIE 2019)	International	Article 7.13.4 of the Code specifies 9 animal-based criteria that are useful indicators of animal welfare. This is comprehensive covering Domains 1–4.	Yes Extensive commercial	No Assessment of the 9 criteria will involve an on-farm visit of long duration and use of farm records. Further, the criteria are based on commercial domestic pig production systems.

^a^In the context of the evaluation of each protocol, comprehensive related to inclusion of indicators of the 4 physical domains of the Five Domains Model and robust related to the indicators being able to be recorded on farm in a consistent and reliable manner

^b^Extensive pig production system with all/some pigs kept in outdoor enclosures with shelter (shown in bold in Supplementary information 1)

All 34 of the publications using the 16 welfare assessment protocols had application to conventional, commercial farms, with the protocols primarily tested for fattening or growing pigs on intensive farms (pigs housed indoors in pens), intensive farms with pigs housed indoors in pens that have outdoor access and organic farms (that meet a range of required standards including that pigs are kept with access to an outdoor area).

For composition of the 16 protocols, 3 were comprised entirely of animal-based indicators (which evaluate the response of or the effect on an animal to assess welfare), 6 entirely of resource-based indicators (which evaluate features of the environment associated with welfare) and management-based indicators (which evaluate management processes and actions of animal owners/attendants), and 7 were a combination of animal-based indicators with resource-based indicators and management-based indicators.

In terms of direct assessment of all four domains (1 to 4) of the FDM, the majority of protocols (10/16) were comprehensive, two were reasonably comprehensive (direct assessment of three domains and indirect/minimal assessment of the remaining domain) and four were not comprehensive.

Among the comprehensive protocols, those involving on-farm assessment of predominantly animal-based indicators provide an in-depth evaluation of pig welfare; however, this evaluation takes up to six hours to complete (such as the Welfare Quality® (WQ) protocol). For those comprised of more resource-based and/or management-based indicators than animal-based, the on-farm time requirement is less but also involves use of farm records and/or use of specific software (Barbari et al. 2008; Bracke et al. 2002; Friedrich et al. 2020a). Protocols focused on iceberg indicators, the animal-based indicators that reflect the consequence of several factors adversely impacting on welfare (eg. body condition, tail damage, mortality rate) (Czycholl et al. 2017b; EFSA Panel on Animal Health and Welfare 2012; Friedrich et al. 2020b), require a shorter time period on-farm. This focus is evident for 4 protocols, of which 1 is comprehensive (EFSA Panel on Animal Health and Welfare 2012) and 3 are not comprehensive (Friedrich et al. 2020b; Pandolfi et al. 2017; Wadepohl et al. 2019), with one reporting inter-observer reliability that demonstrated it is a simple and robust tool for pig welfare assessment (Wadepohl et al. 2019).

Overall, 3 protocols presented evidence, such as inter-observer reliability, as proof that the protocol was a consistent and reliable tool for on-farm assessment of all or the majority of indicators (Friedrich et al. 2020a; Wadepohl et al. 2019; Welfare Quality® 2009). Notably, for the WQ® protocol, this evaluation found specific indicators to be unreliable in two or more of the 18 publications, including bursitis (Alpigiani et al. 2016; Czycholl et al. 2016a, b; Friedrich et al. 2019a; Temple et al. 2013), skin condition (Alpigiani et al. 2016; Temple et al. 2013), the qualitative behavioural assessment (Czycholl et al. 2017, 2016a, b; Friedrich et al. 2019b; Temple et al. 2013) and the human-animal relationship test (Czycholl et al. 2016a; Friedrich et al. 2019b).

To obtain information on the indicators, most of the protocols (10/16) describe use of a questionnaire to obtain data from the manager/owner in person or remotely and 8 describe use of a record sheet or checklist by the assessor to record observations on-farm, with a total of 6 protocols describing use of a questionnaire and a record sheet/checklist in combination during an on-farm visit (Barbari et al. 2008; Ben-Dov et al. 2014; Bracke et al. 2002; Friedrich et al. 2020a, b; Welfare Quality® 2009). All the protocols state or infer that the assessors need to have knowledge of pig management systems, housing and equipment and must be pre-trained in use of the protocol prior to undertaking welfare assessments.

None of the protocols were suitable for direct application on smallholder pig farms in LMIC countries. The most common reasons for this were that the protocol was designed for use in large-scale confined pig housing and management systems, required a farm visit of long duration often including close observation of the pigs, and/or required data from farm records.

### Development of the new framework

Given this finding that no reviewed pig welfare assessment protocol was applicable to smallholder pig settings, we proceeded to develop a preliminary version of a new protocol suitable for welfare assessment of the four physical domains of the FDM in LMIC smallholder pig systems.

Based on the review of existing protocols and considering the various management systems across smallholder pig farms in Africa, Asia and the Pacific, we determined that the new protocol needed to be: (i) suitable for completion during 1–2 hours on-farm, (ii) comprehensive (that is, including indicators of Domains 1 to 4), (iii) comprise of indicators assessed in a straight-forward manner by observation, and (iv) feasible for completion whether pigs are free-roaming or confined by tethering, in outdoor pens/paddocks/yards or in indoor pig sheds.

Of the existing protocols, five protocols with simplified assessment tools requiring less time on-farm, some of which are comprised of iceberg indicators, were chosen to inform the structure for the new protocol and were the basis for selection of resource-, management- and animal-based welfare indicators to include in it. These protocols were Animal Needs Index (Annen et al. 2011), Farm Welfare Index (Barbari et al. 2008), Bien-Être en Élevage de Porcs (BEEP) (Courboulay et al. 2020), Herd Health and Welfare Index (Wadepohl et al. 2019) and Iceberg Indicators (Friedrich et al. 2020b).

Drawing on the strengths and more consistent features of the structure and indicators of these protocols, this new protocol consists of two parts: a questionnaire (Table 6) and an observation record sheet (Tables 7 and 8). These gather information related to welfare indicators that we purposely selected for inclusion to enable assessment on farm of Domains 1–4 and that we refined to be applicable in smallholder pig settings (eg. indicators based on observation, not on farm records as few smallholders keep written records). Considerations in selection of these welfare indicators are discussed in Sect. 3.3 and the definitions and scoring of each indicator are presented in the observation record sheet (Table 7). Prior to implementation of this new protocol, assessors familiar with smallholder pig management systems will need thorough training to be prepared to undertake observations and score indicators in a consistent manner (See Tables 7 and 8).

**Table 6:** Preliminary version of an on-farm welfare assessment protocol designed for use on smallholder pig farms in low-to-middle income countries comprised of a questionnaire and an observation record sheet

Questionnaire	
Name of farmer:	
Breed of pigs:	
Number of adult pigs:	
Number of growing pigs:	
Number of suckling piglets:	
Housing system:	
Type of flooring/substrate:	
Outdoor access:	
Enrichment:	
Feed type:	
Feed storage:	
Mortality rate over the last 12 months for adult pigs:	
Culling rate over the last 12 months for adult pigs:	
Number of sick/injured pigs:	
Biosecurity measures:	
Vaccination status:	.

**Table 7:** Observation record sheet

Domain	Welfare Indicator/ Indicator type (AB/MB/RB)	Score	Classification	Definition
Nutrition	**Water supply/RB**	**2** **0**	**Free access to clean water** **No free access to clean water**	**Clean water is freely accessible from water source/s such as water drinkers, troughs, dams, ponds, rivers.**
	Distance to access water/RB	2 1 0	Within 50 m Between 50–100 m Over 100 m	Average distance that a pig has to walk to access the nearest water source.
	**Feed supply/RB**	**2** **0**	**Access to feed** **No access to feed**	**Feed is accessible either through foraging or feed being provided by farmers. Type/s of feed and feed availability will be determined by time of year.**
Physical environment	**Space allowance/RB**	**2** **0**	**≥1.3 m**^**2**^**/sow** **<1.3 m**^**2**^**/sow**	**Based on measurement of the space/s in which sows are confined and the total number of sows, the space allowance per sow is calculated in m**^**2**^**/sow.** **For free-roaming sows, the space allowance of ≥1.3 m**^**2**^**/sow will apply.**
	Shade/RB	2 0	Access to shade No access to shade	Shade is present (owner provided or naturally occurring) and can be accessed by pigs. Shade sources such as pen roof, man-made canopy, free access to an area underneath or inside a building, shrubs, trees.
	Temperature/RB	2 0	Temperature 15–30 °C Temperature below 15 °C or above 35 °C	Environmental temperature range on day of visit. Temperature range for optimal comfort of growing pigs and adult pigs is 15–30 °C and of newborn piglets is 27–35 °C. (Primary Industries Standing Committee 2008)
	Ammonia levels/RB	2 1 0	<11ppm 11ppm >11ppm	Ammonia level on day of visit on farms with pigs housed indoors 24/7 and measured inside the pig shed/fully enclosed pig pen. Maximum recommended level of 11ppm in completely enclosed pig housing. (Primary Industries Standing Committee 2008)
	Panting/AB	2 0	No panting Evidence of panting	Pigs (excluding piglets) should be observed at rest. Panting is defined as breathing rapidly in short gasps and carried out by breathing through the mouth. Panting is occurring when the Respiratory Rate > 28 breaths/minute in sows. (Welfare Quality® 2009) A score of 0 is allocated when > 10% of pigs on the farm are seen to be panting.
	Huddling/AB	2 0	No huddling Huddling evident	Pigs (excluding piglets) should be observed at rest. Huddling is defined as a pig lying with more than half of its body in contact with another pig. (Welfare Quality® 2009) A score of 0 is allocated when > 10% of pigs on the farm are seen to be huddling.
	Coughing/AB	2 0	No coughing Coughing evident	After making the pig/s stand up, observe for coughing over a 5-minute period.
Health	**Body condition score (BCS)/AB**	**2** **1** **0**	**>50% of pigs with BCS > 2** **>50% of pigs with BCS = 2** **>50% of pigs with BCS < 2**	**BCS scored from 1 to 5 with score categories of 1 (emaciated), 2 (thin), 3 (normal/ideal), 4 (fat), 5 (overly fat).** (Primary Industries Standing Committee 2008)
	Lameness/AB	2 1 0	Normal gait, using all legs Animal is severely lame, minimum weight on the affected limb No weight-bearing on affected limb, or animal unable to walk	Pig must be walking to observe gait.
	Scouring/AB	2 1 0	No liquid manure visible Some liquid manure visible All faeces visible is liquid	Scouring is not assessed by observation of the pigs. Instead it is assessed by observation of liquid faeces on the floor/ground where pigs are present.
	Skin lesions/AB	2 1 0	>50% of pigs with ≤4 lesions visible >50% of pigs with 5–10 lesions visible >50% of pigs with >10 lesions visible	A skin lesion is a scratch/wound > 2 cm in length. Assess by observation of 5 body regions: ears, front (head to back of shoulder), middle (back of shoulder to hindquarters), hindquarters, legs. (Welfare Quality® 2009)
	Tail biting/AB	2 0	No evidence of tail biting Evidence of tail biting	Tail biting is assessed when there is fresh blood visible on the tail, including swelling and signs of infection. The tail can also be missing or have crust apparent
	Faecal egg count/AB	2 0	Negative Positive	Faecal egg count is performed to determine the presence/absence of common internal parasites. If a positive result, provide details on parasite species and eggs/gram of faeces.
	Skin scraping/AB	2 0	Negative Positive	Skin scraping is performed to determine the presence/absence of common external parasites. If a positive result, provide details on parasite species.
Behavioural interactions	**Negative social behaviour/AB**	**2** **1** **0**	**No negative behaviour** **<50% exhibiting negative behaviours** **>50% exhibiting negative behaviours**	**Is defined as an aggressive interaction such as biting or any behaviour that initiates a response from the disturbed animal and includes observation of tail in mouth behaviour.**
	Positive social behaviour/AB	2 1 0	>50% exhibiting positive behaviour <50% exhibiting positive behaviour No positive behaviour	Is defined as sniffing, nosing, licking, and moving gently away from another pig without an aggressive or flight reaction from that individual
	Stereotypic behaviour/AB	2 0	No stereotypic behaviour observed Stereotypic behaviour observed	These are behaviours with no obvious gain or purpose for the animal, including sham chewing, tongue rolling, teeth grinding, bar/trough/drinker biting and floor licking. (Welfare Quality® 2009)
	Environmental investigation/AB	2 0	Evidence of investigation No evidence of investigation	Investigation is defined as sniffing, nosing, licking or chewing features in the environment.
	Enrichment use/AB	2 0	Evidence of using enrichment No evidence of using enrichment	If enrichment is present, evidence of playing with it, including pushing it around with nose, legs or body.
	Fear of humans/AB	2 1 0	>50% of pigs allow assessor to touch between the ears without withdrawal >50% of pigs withdraw initially but then allow assessor to touch between the ears >50% of pigs withdraw and stay withdrawn	Should be assessed early on during the farm visit. Animals must be aware of presence of assessor before initiating assessment of measure.

Measure Type Key: AB = Animal-based measure; MB = Management-based measure; RB = Resource-based measure

Indicators shown in bold are the 5 Critical Indicators

**Table 8:** Summary of welfare indicator scores

Scores	Total number of indicators with score of	% of 23 indicators with score of	Names of Critical Indicator/s that scored zero
0			
1			
2			

Score Key: 0 = Considerable improvement required; 1 = Some improvement required; 2 = No improvement required

### Animal welfare indicators by domain

#### Nutrition

Domain 1 Nutrition is measured by the availability of drinking water and access to feed. Adequate water and feed will ensure that the animals have absence from thirst or hunger leading to a positive mental state. In smallholder production systems nutrition is determined by the availability of feedstuffs which may vary by season/time of the year. For this new protocol, there is a basic evaluation of access to sufficient water and food to sustain life and promote healthy well-being, and the animal-based indicator of body condition score is assessed as it is the easily observable outcome of adequate/inadequate nutrition (recorded under Health).

The questionnaire collects information on feed type/s at the time of visit and on feed storage. The observation sheet records whether there is access to water and to food along with body condition score (under Health). These indicators are recorded for all pigs on the farm, excluding suckling pigs.

#### Physical environment

Domain 2 Physical environment considers the physical and environmental conditions that animals are exposed to which impact on their physical, thermal and respiration comforts.

Physical comforts relate to space allocation and flooring substrate used. The minimum requirements for space allowance differ depending on the housing system, however all pigs must have the space to be able to lie down together without overlapping. The European Union Council Directive states that the total unobstructed floor area available for pregnant sows kept in groups must be at least 1.3 m^2^ (Council of the European Union 2009). Similarly, the Model Code of Practice for the Welfare of Animals, Pigs states that sows in group housing must have a minimum space allowance of 1.4 m^2^ (Primary Industries Standing Committee 2008).

Thermal comforts relate to the availability of shade and shelter as well as the temperature of the environment that pigs are kept in. The indicators selected for assessment of thermal comfort include panting, huddling, environmental temperature and shade availability (either man-made or shade from trees in the environment). For panting and huddling, all pigs are assessed (excluding suckling piglets) and if more than 10% are panting or huddling then thermal stress is present.

Respiration comforts relate to the ability of the environment to dissipate foul smells, particularly, ammonia level, which is assessed to determine whether the air is is suitable for breathing/respiration without harm to the respiratory tract. Instrument measurement of ammonia levels will apply only on farms where pigs are housed indoors. For other farms, assessor will score ammonia based on smell as present/absent.

The questionnaire collects information on housing system, outdoor access, type of flooring substrate and presence of enrichment. The observation sheet records space allocation for sows and shade, environmental temperature, ammonia level, panting, huddling and coughing for all pigs on the farm (with the exception of suckling piglets for panting and huddling).

#### Health

Domain 3 Health considers the physical condition of animals in relation to disease, injury, body condition and fitness in order to inform actions to protect against disease and injury, and to enhance mobility and fitness. Good health is indicated by the absence of disease and injury, thus the following animal-based indicators were selected for inclusion in the protocol.

Body condition score (BCS) is scored from 1 (emaciated) to 5 (overly fat) as stated in the Model Code of Practice for the Welfare of Animals, Pigs (Primary Industries Standing Committee 2008). On each farm the number of pigs with BCS of <2, 2 and >2 will be recorded in order to assess the overall welfare of pigs on the farm in this criterion.

Lameness score is determined by observation of the pigs when walking and categorized as normal gait, severely lame with minimal weight bearing on affected limb, and unable to walk.

Scouring, when faeces become more fluid in consistency than normal, is a sign of enteric disorders and can be used to monitor the level of unwell pigs on a farm. For this protocol, it will be determined by observation of liquid faeces on the floor/ground where pigs are present on the farm.

Skin lesions are defined as wounds on the body which to standardize assessment under the WQ® protocol are recorded by observation of specified regions of the pig’s body (ears, front, middle, hindquarters, legs) and weighted for severity (based on length, depth, extent of blood) (Welfare Quality® 2009). To simplify assessment for this protocol, a scratch/wound longer than two centimeters will be considered a lesion and the number of lesions totaled after observation of the five body regions on all pigs on the farm.

Tail biting is a measure assessed when pigs are standing and is a measure of damage to the tail ranging from superficial wounds to absence of the tail. For this protocol, evidence or not of tail biting will be recorded based on observation of any evidence of tail biting on the farm.

Assessment of parasitic burden among pigs on a farm must consider the presence and extent of internal parasites (via faecal egg count) and external parasites (via skin scraping) that are common in the geographic region where a farm is located. For this protocol, it is proposed that faecal egg count and skin scraping be conducted on 10% of the grower pigs and of the adult pigs on a farm irrespective of herd size to provide initial evidence of parasite absence or presence.

The questionnaire collects information on mortality rate, culling rate, number of sick/injured animals on the day of visit and vaccination status. The observation sheet records BCS, lameness, scouring, skin lesions, tail biting and parasitic burden for all pigs on the farm (with the exception of suckling piglets for parasitic burden).

#### Behavioural interactions

Domain 4 Behavioural interactions are the interactions of an animal with the environment, other animals and humans. A set of indicators were included in the protocol to ensure complete evidence of hindered or enabled agency to actively engage with the external physical, biological and social environment (Mellor et al. 2020).

Time of day and length of observation must be specified for these indicators to provide equivalent opportunity to witness behaviours. A minimum of 10 minutes observation time should be set during the morning when pigs are more active but not including or within 10 minutes of feeding time as this may skew natural behaviours.

Negative and positive social behaviour relate to interactions with other pigs on farm. Negative behaviours include aggressive behaviours such as biting or head butting while positive behaviours include sniffing, nosing, licking, and moving past without initiating aggression or a flight reaction.

Stereotypic behaviours are sequences of invariant motor acts with no obvious gain or purpose for the animal. In pigs these include sham chewing, tongue rolling, teeth grinding, bar/trough/drinker biting and floor licking.

Environmental investigation includes any investigation of the pigs’ surroundings irrespective of housing system. This investigation includes sniffing, nosing, licking or chewing of pen features for penned pigs, of any objects within tethering range for tethered pigs, and these behaviours along with rooting, nesting and other interactions with environmental features for extensively housed/yarded and free-range pigs.

Evaluation of enrichment use requires the presence of enrichment in the housing system (eg straw, toys, fallen branches) and time to observe whether the enrichment stimulates pigs to play or investigate these objects in their environment.

The human-animal relationship is a major contributing factor to the overall welfare of pigs. Assessment needs to be performed early in the farm visit by the assessor directly interacting with pigs on the farm. Ability of the assessor to touch a pig without a withdrawal response demonstrates a positive human-animal relationship.

These six behaviour indicators are recorded on the observation record sheet based on observation of all growing pigs and adult pigs on a farm.

### Questionnaire

The questionnaire presented in Table 6 will be completed during an interview with the farmer either in-person, on-farm or via phone call that will take place prior to the assessor conducting observations on-farm. It is comprised of 15 questions to obtain data on smallholder pig herd demographics, on biosecurity practices, and on resource-based and management-based welfare indicators (listed in Sect. 3.3). In situations where the farmer does not routinely keep written herd records of pig herd size and composition along with pig deaths or disposal of cull sows, this will have to be noted by the assessor and the farmer requested to recall the number of pigs raised, pig numbers over the last 12 months along with pig deaths and culling/disposal of cull sows/boars.

The biosecurity practices question aims to document the actions taken when new pigs are introduced to the pig herd and when pigs are moved between farms (including borrowing a boar for mating by farmers with no boar) or to markets in the region. This informs understanding of the risk for disease introduction to the herd with subsequent adverse impact on pig welfare, along with the potential for disease outbreaks to arise from movements of pigs in the region. Further, pig vaccination status for infectious diseases prominent in the geographic region is recorded.

### Observation record sheet

On completion of the interview, the trained assessor will undertake observations to complete the observation record sheet. It is comprised of 23 items to obtain data on 7 resource-based welfare indicators and 16 animal-based welfare indicators, with definitions and scoring shown in Table 7.

For easy assessment, the welfare indicators are scored on a three-point scale, with each score designation reflecting the following: zero - considerable improvement required; one - some improvement required; two - no improvement required. The number of indicators with a score of zero, one and two are totaled at the bottom of the sheet and the proportion of the 23 indicators in each score category calculated (Table 8). Welfare indicators with a score of zero need prompt action to improve/rectify pig welfare status and are to be addressed before indicators with a score of one.

Among the 23 indicators, five indicators are designated as critical indicators and a farm that scores a zero for one or more of these critical indicators is automatically categorised as in urgent need of immediate intervention to improve pig welfare. The critical indicators are: 1) Access to water, 2) Access to feed, 3) BCS (three indicators that relate to meeting the survival needs of a healthy animal), 4) Space allowance (relates to adequate space to exhibit normal behaviours), 5) Negative social behaviour (relates to occurrence of behaviours that cause injury/harm to pigs). These critical indicators are to be given priority for assessment, and for immediate, targeted action when a farm is categorized as in urgent need of immediate intervention followed by reassessment post-intervention.

## Discussion

Through a review of pig welfare assessment protocols in use around the world, we determined that no existing protocol was directly applicable to the context of smallholder pig farms in LMIC. Thus, we took the initiative to develop a preliminary version of a new protocol utilizing indicators suitable to assess the overall welfare of pigs on-farm in LMIC smallholder pig systems.

The intended purpose for this smallholder pig specific welfare assessment protocol is to conduct a context-appropriate assessment of pig welfare that identifies and priorities areas for improvement whilst not making any judgements about husbandry conditions. Recently, a study describing the welfare issues in Ugandan pig farms stated that all aspects of pig welfare are compromised and that immediate interventions are needed (Dione et al. 2022). It reported welfare concerns for pigs found to be exposed to undernutrition, dirty water, high mortality, physical injuries, poor housing and health challenges, then put forward simple interventions that could enhance both farmer livelihoods and the welfare of their pigs. Use on smallholder pig farms in Uganda by Dione et al. (2022) of many welfare indicators included in this welfare protocol strengthens our conviction that it is feasible for use in a variety of smallholder contexts and will provide reliable data on pig welfare status.

For the domain of nutrition, water and feed access along with distance to access water and body condition score were selected as the simplest indicators by which to assess the basic nutritional needs of pigs without obtaining detail on diet composition, which cannot be known for free-roaming pigs. Access to feed and to water are both critical indicators for nutrition because pigs must have daily access to feed and water to maintain their health and meet their physiological requirements (Primary Industries Standing Committee 2008). In addition to these indicators highlighting current adequate/inadequate access to nutrition, body condition score is a readily observed measure of longer term adequate/inadequate nutrition. Thus, the designation of BCS as a critical measure in this welfare protocol reflects the fundamental link between adequate nutrition and sound health. When chronic undernutrition is obvious from emaciated body condition, an in-depth investigation into the pig diet could be performed to identify the feedstuffs available each season and to provide advice to the community on formulation of nutritionally adequate pig diets using locally available feedstuffs.

For the domain of physical environment, space allowance was determined to be the critical measure, with shade, panting, huddling, coughing, temperature, and ammonia levels all providing another level of assessment. Space allocation directly relates to stocking density in terms of floor area per pig. The link between space allocation and tail biting emphasizes the importance of the availability of adequate space (Sandøe et al. 2020) along with the evidence that crowding results in reduced overall performance including reduced growth (Street and Gonyou 2008). Ability to measure ammonia levels depends on the type of pig housing and may not be feasible in extensive free-range systems, or even applicable. However, it is an important indicator to retain when smallholder systems consist of different types of housing systems (indoor, enclosed pens vs. extensive, outdoor) and the impact of high ammonia air quality has such a significant impact on welfare and production (Banhazi et al. 2008).

For the domain of health, body condition score is the critical measure with lameness, scouring, skin lesions, tail biting, faecal egg count and skin scraping as further indicators that together provide a holistic view of this welfare domain. Health is directly influenced by the setting in which an animal lives and for smallholder pigs this varies widely in terms of geoclimatic conditions and management system (from intensive pens to free roaming). The extent to which nutrition and the physical environment of these pigs is under the control of the smallholder farmer differs substantially between regions and farms; even within regions, the resources farmers provide for their pigs differs substantially (Dione et al. 2022). This wide variation in farmer control is a unique/distinctive feature of smallholder systems compared to commercial pig systems. Gold standard evaluation of body condition score involves observation and palpation of specific anatomical areas of the pig (Primary Industries Standing Committee 2008). While palpation of free-roaming pigs may not be possible, proximity permitting whole body view to score health indicators can be facilitated by visiting at the time of day the pigs are usually at the residence. However, faecal egg count and skin scraping indicators require pig restraint and availability of a microscope and other equipment which will limit their application to more urban contexts with confined pigs.

Biosecurity, the management practices that minimise the risk of pathogen introduction to and spread on a farm, are often reported as absent or minimal on smallholder farms. For example, though a national import biosecurity plan often exists to prevent disease entry to the country, animal movement control may not be well regulated and most smallholder farms do not have a biosecurity protocol for trading pigs between farms (Barnes et al. 2020; Maes et al. 2020; Mutua and Dione 2021; Woonwong et al. 2020). Thus, poor biosecurity is a contributor to adverse health impacts on smallholder pig welfare. The implementation of basic biosecurity measures, such as hand hygiene, dedicated personal protective gear (boots and overalls) and a barrier fence surrounding pig pens piloted to help avoid African Swine Fever (ASF) introduction in Timor Leste, also offer promise as contributors to improved pig welfare under the health domain (Barnes et al. 2020).

For the behavioural interactions domain, negative social behaviour was determined to be the critical measure, followed by positive social behaviour, stereotypic behaviour, environment investigation, enrichment use, and fear of humans. There are well-documented benefits from enabling pigs to express behaviours such as exploring the environment, which is proven to reduce aggression within a herd and reduce skin lesions (Godyń et al. 2019) and is stimulated by the presence of enrichment. In contrast, stereotypic behaviours are indicators of frustration and the attempts of an animal to cope within the confines of an environment that is having an adverse impact on welfare. Although there may be challenges for observing stereotypies on smallholder farms, such as the small number of pigs and the novelty of an observer’s presence which may make accurate detection by the observer difficult, we have included stereotypic behaviour to start investigation of in these production systems. Compared to individually penned pigs on commercial farms, it may be that less strictly confined smallholder pigs exhibit fewer stereotypic behaviours due to interactions with a changing environment. Similarly, though we anticipate challenge in measuring fear of humans due to variation in pig ease with close observer proximity, it is included to ensure consideration in future protocol refinement.

Pigs are important for rural households in many LMIC countries. As such improving the welfare of these animals should be investigated as one avenue to improve pig raising and its contribution to household income (De Almeida et al. 2021). In Vietnam, though few pork consumers surveyed had heard of the term ‘animal welfare’, many stated that pigs should be kept in hygienic conditions and some that pigs should have room to move around and exhibit natural behaviours (Le et al. 2022). Fundamental to achieving provision of these basic needs for pig welfare is knowledge and awareness along with farmer agency to provide for the needs. Insufficient knowledge regarding many aspects of pig diseases and pig husbandry practices has been identified in multiple smallholder contexts along with the finding that resource-poor rural pig keeping is dominated by free-roaming pigs (Chilundo et al. 2020b, De Almeida et al. 2021; Dione et al. 2017, 2014). However; knowledge alone is not going to lead to practice change, with the reasons for not providing resources to pigs being multifactorial. For example, the non-provision of water to pigs has been credited to lack of farmer knowledge about the importance of water, the scarcity of water for human consumption, and the practice of adding water to food rather than providing water alone (Amben et al. 2017; Braae et al. 2016; Chilundo et al. 2020a). Inadequate feeding is also common, with famers in Timor-Leste reporting severe shortages of available feed for people and pigs during specific seasons (De Almeida et al. 2021). While there is increasing attention from authorities on animal welfare, knowledge and awareness about animal welfare is still lacking across smallholder pig production systems (Braae et al. 2016; Smith et al. 2021). Addressing this lack of public awareness through community wide education and training is perceived to be important for animal welfare to be prioritized by smallholder farmers, given that currently farmers are willing to make changes only if they have external help and the potential for incentives (Chilundo et al. 2020a; Mutua et al. 2020; Smith et al. 2021). It is reasonable in resource-poor settings, that livelihoods providing secure household income need to be established first before pig owners will be able to invest in better animal health and welfare (Okello et al. 2020). Thus, financial constraints are a major limiting factor to improvements in pig management that will achieve better welfare (Cronin et al. 2014; Mbuthia et al. 2015; Zewdie et al. 2020). Understanding the welfare status of pigs in smallholder systems is important to see where win-win improvements for pigs and for owners can be made. Through a lens of animal welfare, improvements that address welfare issues can be made, while recognizing and not impinging on the welfare benefits of existing management.

This preliminary protocol needs to be piloted by trained observers on smallholder farms raising pigs under different management systems to test the utility of selected indicators and inform modification to improve ease of use. It is possible, that reliance on five existing protocols for large-scale commercial farms as a basis for protocol development, brings inherent as yet unforeseen limitations to application on smallholder farms that will only be realized during pilot tests. It is important for field validation to occur with pilot tests designed to assess intra- and inter-observer reliability and careful consideration given to the preparatory training of observers to ensure consistent protocols for observations and sample collection and measurement (such as for faecal egg count and skin scrapings). Along with pilot testing, evaluation against regional and national pig welfare guidelines established in recent years (for example, ASEAN (2023)) and longer standing guidelines published in languages other than English not captured in this scoping review, will inform improvements to the protocol. Given the diversity in environmental, economic, and management conditions across smallholder contexts, these future pilots and evaluations will identify some limitations and provide refinements to some indicators to enable better application to specific contexts. The diversity of contexts also provides a challenge to comparability of the results for farms in different settings. This is an area that warrants further thought and it may be that a small set of protocols, each one tailored to a specific smallholder management type and potentially also continent or world region due to geoclimatic differences across Africa, Asia and Central/South America, will emerge.

A protocol suitable for farmer use on farm is the aspirational goal. Beyond design of context-appropriate protocol/s, protocol adoption by farmers will require improved understanding of pig welfare; low-cost, context-appropriate practices to address the most critical welfare constraints; and farmer realization of sustained livelihood benefit from changing management practices. We forsee achievement of this ambitious goal to be realized by integrating welfare in community-based livelihood enhancement initiatives, with measures of profit, production, biosecurity and welfare providing evidence of impact. The presentation of this preliminary pig welfare assessment protocol is an essential initial step to this end.

## Conclusion

The 16 pig welfare assessment protocols reviewed provided valuable insight into measuring pig welfare on farms, but none was directly applicable to smallholder contexts in LMIC. The preliminary new protocol presented is simpler than the existing protocols and comprised of indicators applicable to smallholder pig farms. While it requires pilot implementation and refinement to ensure it is a suitable and reliable protocol, its application in research and extension will provide new knowledge about the welfare of pigs in different smallholder production systems, and integration of welfare into assessments of production, biosecurity and livelihood impacts will strengthen sustainable development initiatives for smallholder communities to benefit people and their pigs.

## Electronic supplementary material

Below is the link to the electronic supplementary material.

Supplementary Material 1

## Data Availability

This manuscript has no associated data.
