# Supplementary material for: Framing a pig welfare assessment protocol suitable for smallholder settings in low-to-middle income countries
Source: Trop Anim Health Prod. 2026 Jun 12;58(5):337. doi: 10.1007/s11250-026-05131-5 (PMC13263190; doi:10.1007/s11250-026-05131-5)
Supplement: Supplementary file 1 — Supplementary Material 1 [file 11250_2026_5131_MOESM1_ESM.docx]

**Supplementary information 1.** Details of the 34 publications identified in 2021 applying one of 16 welfare assessment protocols to assess welfare in one or more pig production system.

^a^ Extensive pig production system with all/some pigs kept in outdoor enclosures with shelter are shown in bold. Non-extensive pig production systems in which pigs are kept indoors with access to an outdoor area but are not classified as organic farms are shown in italics.

^b^ Intensive refers to large-scale farms under conventional management with pigs housed indoors in pens.

^c^ Organic meeting a range of required standards including that animals are kept with access to an outdoor area.

^d^ Comparison of the protocol against the Five Domains Model as the gold standard to ascertain whether the protocol was comprehensive (that is, including measures of the 4 physical domains of the FDM) and robust (that is, including measures that can be recorded on farm in a consistent and reliable manner).

^e^ Rustic production units in Mexico that are medium-scale farms with all sows kept in pens but may have varied housing to confine other categories of pigs and may have other livestock on farm.

| Welfare assessment protocol | Publication | Country research conducted | Brief outline on focus of the paper | Type of production system^a^ | Comparison to Five Domains Model to ascertain whether Comprehensive and Robust^d^ | Considerations regarding feasibility for implementation on smallholder pig farms in low-to-middle income countries |
| --- | --- | --- | --- | --- | --- | --- |
| Welfare Quality® (WQ) protocol | Alpigiani et al, 2016  (Alpigiani et al. 2016) | United Kingdom | Finishing pigs assessed for animal-, resource- and management-based welfare measures to evaluate association with receptiveness to two enteric pathogens. | Finishing pigs  Intensive^b^  **Semi-free range**  *Organic^c^* | Assessed Domains 1-4 on farm by applying a selection of WQ measures.  Animal-based measures were found to provide the most reliable, irrespective of farming system.  The individual animal measures of bursitis, scouring and skin condition could not be recorded reliably. | Included Domain 3 measures that require close, uninterrupted observation of pigs.  Mortality % requires record of total pigs and total died during last 12 months.  Included Domain 3 measures of stomach and viscera assessed at slaughter off farm. |
|  | Andronie et al, 2014  (Andronie et al. 2014) | Romania | Fattening pigs raised intensively monitored for sanitary and behavioural status | Fattening pigs  Intensive^b^ | Assessed Domains 1-4 on farm by applying all WQ measures (except castration and tail docking). | Included Domain 3 measures that require close, uninterrupted observation of pigs.  Mortality % requires records of total pigs and of total died during last 12 months.  Qualitative Behaviour Assessment (QBA) scoring 20 descriptors using a visual analogue scale is complex and involves multiple observation points. |
|  | Czycholl et al, 2017 (Czycholl et al. 2017) | Germany | Assessed the reliability on-farm of the Qualitative Behavioural Assessment (QBA) included in the Welfare Quality protocol for growing pigs | Growing pigs  Intensive^b^ | Assessed Domain 4 on farm by applying QBA only.  Fixed-list QBA was found to have unsatisfactory reliability for on-farm application compared to good reliability for application based on video footage. | Qualitative Behaviour Assessment (QBA) scoring 20 descriptors using a visual analogue scale is complex and involves multiple observation points. |
|  | Czycholl et al, 2016 (Czycholl et al. 2016) | Germany | Assessed interobserver reliability of the Welfare Quality® protocol | Growing pigs  Intensive^b^ | Assessed Domains 1-4 on farm by applying all WQ measures (except castration and tail docking).  Interobserver reliability was very low for QBA, and the individual animal measure of bursitis. But found to be good for behavioural observations and other individual animal measures. | Included Domain 3 measures that require close, uninterrupted observation of pigs*.*Mortality % requires records of total pigs and of total died during last 12 months.  Qualitative Behaviour Assessment (QBA) scoring 20 descriptors using a visual analogue scale is complex and involves multiple observation points. |
|  | Czycholl et al, 2016a (Czycholl et al. 2016a) | Germany | Assessed the feasibility and test-retest reliability of the Welfare Quality® protocol | Growing pigs  Intensive^b^  *Intensive with outdoor access to a fully slatted area.* | Assessed Domains 1-4 on farm by applying all WQ measures (except castration and tail docking).  Reliability between farm visits was unsatisfactory for QBA and human-animal relationship test (HAR), but good for behavioural observations.  Individual animal measures had acceptable agreement except for bursitis, manure on the body, coughing and sneezing. | Full assessment required up to 6 hours.  Included Domain 3 measures that require close, uninterrupted observation of pigs.  Farm and slaughterhouse records were required eg for mortality and pleurisy.  Qualitative Behaviour Assessment (QBA) scoring 20 descriptors using a visual analogue scale is complex and involves multiple observation points. |
|  | Czycholl et al, 2017a (Czycholl et al. 2017a) | Germany | Assessment of the multi-criteria evaluation model included in the Welfare Quality® protocol for growing pigs in which the animal-based measures were aggregated to criteria, then to principles and finally to an overall farm welfare score. | Growing pigs  Intensive^b^  *Intensive with outdoor access to a fully slatted area.* | Assessed Domains 1-4 on farm by applying all WQ measures.  Several issues were identified that require further development of the aggregation system to ensure accuracy, reliability and correct interpretation of data.  Evidence that some specific measures might be  iceberg indicators, that is, a measure which indicates more than one welfare problem. | Time consuming and complicated assessment involving extensive pig observation, farm and slaughterhouse records and aggregation of measure scores using an algorithm. |
|  | Czycholl et al, 2018 (Czycholl et al. 2018) | Germany | Assessment of the reliability of the multi-criteria evaluation model included in the Welfare Quality® protocol for growing pigs involving aggregation of animal-based measures to criteria, to principles and then to an overall welfare score. | Growing pigs  Intensive^b^  *Intensive with outdoor access to a fully slatted area.* | Assessed Domains 1-4 on farm by applying all WQ measures.  Insufficient repeatability on several criteria and two principles due to difficulty with assessment of some measures. This highlights the importance of absolutely reliable baseline measures.  Further, aggregation system shown to be partially incorrect and in need of correction. | Time consuming and complicated assessment involving extensive pig observation, farm and slaughterhouse records and aggregation of measure scores using an algorithm. |
|  | Dippel et al, 2014(Dippel et al. 2014) | 6 European Union countries (Austria, Denmark, France, Germany, Italy, Sweden) | This paper describes the health and welfare of sows and of suckling and weaned piglets from 101 organic pig farms across six EU countries, using selected animal-based parameters from the Welfare Quality® protocol. | Sows  Suckling and weaned piglets  *Organic^c^* | Assessed Domains 1-3 on farm by applying a selection of WQ measures.  Satisfactory inter-observer agreement was not achieved for all measures showing that intensive training and inter-observer agreement testing is needed. | More feasible because a comparatively small set of animal-based measures based on observation but some measures require close, uninterrupted observation of the pigs |
|  | Friedrich et al, 2019 (Friedrich et al. 2019) | Germany | Tested the consistency over time of the indicators included in the Welfare Quality® protocol, focusing on the welfare principle ‘appropriate behaviour’ - Qualitative Behavioural Assessment (QBA), Behaviour observations, Human-Animal Relationship test (HAR), Stereotypies (ST). | Sows and piglets  Intensive^b^  *Organic^c^* | Presents assessment of Domain 4 on farm – QBA, Behaviour observations, HAR, ST.  Poor test-retest reliability for QBA, HAR.  Acceptable reliability for the majority of Behaviour observations (except use of enrichment, other active behaviour), and for Stereotypies. | Qualitative Behaviour Assessment (QBA) scoring 20 descriptors using a visual analogue scale is complex and involves multiple observation points.  Some measures require close observation of the pigs. |
|  | Friedrich et al, 2019a (Friedrich et al. 2019a) | Germany | Tested the feasibility and on-farm test–retest reliability of the Welfare Quality® protocol focusing on the principles; good feeding, good housing, and good health. | Sows and piglets  Intensive^b^  *Organic^c^* | Presents assessment of Domains 1-3 on farm by applying all WQ measures.  The majority of measures achieved acceptable reliability showing WQ assessment protocol to be a reliable approach to assess sow and piglet welfare.  Poor test-retest reliability for body condition score, bursitis, panting, huddling and a few health measures. | Some measures require close observation of the pigs. |
|  | Losada-Espinosa et al, 2017  (Losada-Espinosa et al. 2017) | Mexico | Assessed welfare of pigs in rustic and technified production systems using the Welfare Quality® protocol for pigs in Mexico.  Findings included that health and behaviour measures are sensitive to changes in the housing and management of pigs. Conclusion that high occurrence of health and behaviour problems on technified units are an indicator of poor welfare. | Sow and piglets  Fattening pigs  All commercial units with confined pigs.  Rustic production units^e^  Technified production units/ Intensive^b^ | Presents assessment of Domains 1-4 on farm by applying all WQ measures.  Researchers conclude that WQ protocol is a useful tool with reliable indicators (criteria) for sustainability assessments. | Researchers conclude WQ protocol feasible for confined pigs noting all units had sows kept in pens.  However, the full WQ is a time consuming and complicated assessment involving extensive pig observation, farm records and aggregation of measure scores. |
|  | Martin et al, 2017  (Martin et al. 2017) | Germany | Validated an alternative multi-criteria evaluation system (using multi-attribute utility theory MAUT) to assess animal welfare of growing pigs on farm based on the Welfare Quality® protocol.  In this, measures are aggregated into criteria, criteria into principles and principles into an overall assessment. | Growing pigs  Intensive^b^ | Assessed Domains 1-4 on farm by applying all WQ measures.  Aggregation results at criteria and principle levels similar to that of Czycholl et al. with more flexible and transparent approach. | Time consuming and complicated assessment involving extensive pig observation, farm and slaughterhouse records and aggregation of measure scores. |
|  | Munsterhjelm et al, 2015  (Munsterhjelm et al. 2015) | Finland | Utilised the Welfare Quality® protocol to assess fattening pigs, sow, and suckling pigs on Finnish farms with Principal Component Analysis (PCA) applied to identify distinct types of welfare problems and QBA descriptors analysed to identify distinct mood types. | Sow and piglets  Growing pigs  Intensive^b^ | Assessed Domains 1-4 on farm by applying all WQ measures.  PCA identified shortlists of animal-based measures with decent to good internal consistency to describe distinct types of welfare problems in growing pigs and in sows. | Assessment involving extensive pig observation plus farm and slaughterhouse records.  Potentially the shortlists of animal-based measures identified by PCA to describe welfare problem types if proven to have strong relationships with environmental or other features may be useful for time-efficient assessment of animal welfare. |
|  | Scott et al, 2009  (Scott et al. 2009) | United Kingdom  Netherlands | Monitoring system developed for sows and piglets using predominantly animal-based measures of behaviour, health, and physiology to assess welfare implemented in diverse production systems. | Sow and piglets  Intensive indoor including farms with sows housed in stalls & housed in groups  *Organic^c^* | Assessed Domains 1-4 on farm by applying WQ measures defined in 2007.  Use of predominantly animal-based measures provides the basis for a useful welfare assessment system.  However, some measures were impractical and could not be recorded for sows kept in large groups or in outdoor settings. | Time consuming taking 4-5 hours to complete.  Some animal-level measures were impractical on outdoor farms, where close proximity to sows for observation was not possible or weather conditions can impede access for observation. |
|  | Temple et al, 2012  (Temple et al. 2012) | France  Spain | Compared the health of growing pigs in five different production systems in France and Spain using measures provided by the Welfare Quality® protocol. | Growing pigs  5 different production systems  Intensive^b^  Intensive straw-bedded  Intensive Iberian  **Extensive Iberian**  **Extensive Mallorcan Black** | Assessed Domain 3 on farm by applying WQ measures defined in 2008.  No comment on issues/problems with consistent & reliable assessment in this study aside from low inter-observer reliability for skin conditions related to 1 observer. | Health measures appear feasible to observe and record across the 5 farm types. Training of observers was emphasised. |
|  | Temple et al, 2011  (Temple et al. 2011) | Spain | Validation of the Welfare Quality® protocol for assessing welfare of growing pigs kept on farms. | Growing pigs  Intensive^b^ | Assessed Domains 1-4 on farm by applying WQ measures defined in 2007, including QBA and HAR test.  Good feeding, housing, and health measures were scored at the pen- or individual-level using a 3-point scale ranging from 0 to 2.  Protocol easy to perform under commercial conditions with little input needed from farmer but average time to complete was 6.3 hours. | Time consuming taking on average 6.3 hours to complete.  Authors state modification needed for use on extensive farms.  Qualitative Behaviour Assessment (QBA) scoring 20 descriptors using a visual analogue scale is complex and involves multiple observation points |
|  | Temple et al, 2013  (Temple et al. 2013) | Spain | Test–retest reliability of quantitative and qualitative animal-based measures included in the Welfare Quality® protocol on a sample of 15 intensive conventional growing pig farms. | Growing pigs  Intensive^b^ | Assessed Domains 1-4 on farm by applying WQ measures defined in 2009, including QBA and HAR test.  Reliability between farm visits was unsatisfactory for QBA and low for bursitis, skin condition and positive social behaviour. | Time consuming and complicated assessment involving extensive pig observation.  Qualitative Behaviour Assessment (QBA) scoring 20 descriptors using a visual analogue scale is complex and involves multiple observation points. |
|  | van Staaveren et al, 2018  (van Staaveren et al. 2018) | Ireland | Assessed welfare in weaner and finisher stages using modified WQ protocol on 31 Irish pig farms | Weaner pigs  Fattening pigs  Intensive farrow-to-finish farms | Assessed Domains 1-3 by applying a modified version of the WQ protocol to determine the prevalence of welfare problems.  No comment on consistency or reliability of measures in this study. | All measures appear feasible by observation from outside pen similar to stockperson on farm but do require observation of each pig. |
| Animal Welfare Indicators: Practical Guide—Pigs Kuratorium für Technik und Bauwesen in der Landwirtschaft e.V. (KTBL) | Friedrich et al, 2020a  (Friedrich et al. 2020a) | Germany | Comparison of sow and piglet assessments using the Welfare Quality® protocol and the Animal Welfare Indicators: Practical Guide—Pigs Kuratorium für Technik und Bauwesen in der Landwirtschaft e.V. (KTBL). The less detailed KTBL protocol based on the WQ protocol was introduced in Germany for farm self-inspection. | Sows  Piglets  Intensive^b^  *Organic^c^* | KTBL for sows, 18 measures assessed Domains 1-4 on farm.  KTBL for piglets, 6 measures assessed Domains 1-3 on farm (equivalent domain coverage as WQ protocol for piglets).  KTBL has fewer measures in total than WQ and a higher proportion of management-based indicators plus litter-level measures for piglets rather than animal-level.  Acceptable reliability given equivalent performance on measures the same as the WQ protocol. | Reduced number of measures enhances the feasibility in terms of time requirement and complexity, but the inclusion of management-based indicators means farm records are required.  Also, information on animal behaviour is more limited. |
|  | Pfeifer et al, 2020  (Pfeifer et al. 2020) | Germany | Investigated the acceptance and feasibility of the KTBL protocol for the animal welfare assessment of fattening pigs from the perspective of livestock farmers. | Fattening pigs  Intensive^b^  *Intensive with outdoor access* | For fattening pigs, assessed Domains 1-3 on farm.  13 indicators  KTBL has fewer measures in total and a higher proportion of management-based indicators than WQ.  No evaluation of tool consistency or reliability.  Majority of farmers agreed 11 indicators were acceptable for assessment of fattening pig welfare (except faecal soiling, tail length), and agreed on feasibility of 12 indicators (except skin lesions). | Reduced number of measures enhances the feasibility in terms of time requirement and complexity, but the inclusion of management-based indicators means farm records are required and there is no consideration of Domain 4 Appropriate behaviour. |
| Iceberg indicators protocol | Friedrich et al, 2020  (Friedrich et al. 2020) | Germany | Proposes a set of ‘iceberg’ indicators for welfare assessment of sows and of piglets based on Hierarchical Component Model analysis of farm data for the Welfare Quality® protocol and the KTLB protocol.  The proposed set of indicators identified for sows includes positive behaviour, stereotypies, frothy saliva, mortality, metritis, shoulder sores, panting.  The proposed set of indicators identified for piglets includes carpal joint lesions, undersized animals, mortality, sneezing. | Sows  Piglets  Intensive^b^  *Organic^c^* | Proposed set for sows, assesses Domains 2-4 (2: shoulder sores, panting; 3: mortality, metritis; 4: positive behaviour, stereotypies, frothy saliva).  Proposed set for piglets, assesses Domains 1&3 (1: carpal joint lesions, undersized animals; 3: mortality, sneezing).  These proposed protocols have fewer measures in total.  Evaluation of reliability of proposed protocols stated to be required to ensure these are objective assessment tools. | Reduced number of measures enhances the feasibility in terms of time requirement and complexity, but the inclusion of management-based indicators means farm records are required. |
| Animal Needs Index | Annen D et al, 2011  (Annen et al. 2011) | Austria  Germany | Conduct of an inventory including comparison of existing private and government animal welfare certification schemes in Austria and Germany followed by development of the Animal Needs Index to evaluate animal welfare and conduct of assessments on fattening pig farms in Austria. | Fattening pigs  Intensive^b^  *Organic^c^*  **Extensive** | Five components that assessed Domains 2-4 and indirectly Domain 1 with a focus on environmental and management-based measures used to control legislative and certification requirements.  1.Possibility of mobility  2.Social interaction  3.Condition of flooring for lying, standing, and walking  4.Climatization (light, air, noise)  5.Intensity or quality of human care.  No comment on consistency or reliability of measures. | It is a simplified assessment tool with consideration for extensive management systems but has multiple measures that require data from farm records. |
| Farm Welfare Index | Barbari et al, 2008  (Barbari et al. 2008) | Italy | Development and components of the Farm Welfare Index used to assess welfare on 80 pig farms located in Northern Italy. | Farrow-to-fattening  Intensive^b^ | Assessed Domains 1-4 on farm to assign a welfare index to a farm (increasing level of welfare from 1 to 6).  The index is based on assessment of 3 categories: general data, buildings, and pig categories (mating/pregnancy, farrowing/lactation, weaning, fattening/breeding).  No comment on consistency or reliability of measures. | It is a simplified assessment tool based predominantly on inspector observation over a 2-4 hour farm visit (depending on farm size and number of buildings) and use of index software. |
| Guidelines for Swine Keeping | Ben-Dov et al, 2014 (Ben-Dov et al. 2014) | Israel | Compared the Guidelines for Swine Keeping against the European Council Directive 2008/120/EC (minimum standards for the protection of pigs) and reports application of the Guidelines on the 24 pig farms in Israel. | Farrow-to-fattening  Intensive^b^ | 22 guidelines that assessed Domains 1-3 and to a minimal extent Domain 4, with emphasis on meeting stated requirements for building/facility, staff, equipment, feed, water.  No comment on consistency or reliability of guidelines but given present/absent nature of guidelines, there is little ambiguity. | The guidelines involve observation during a relatively short on farm visit but are heavily based on intensive indoors management system with limited consideration of pig behaviour. |
| German National Assessment Catalogue for Animal Husbandry (NACAH) | Bergschmidt & Schrader, 2009  (Bergschmidt and Schrader 2009) | Germany | Applied and adapted the National Assessment Catalogue for Animal Husbandry to evaluate the Farm Investment Scheme on animal behaviour for fattening pigs. | Fattening pigs  Intensive^b^  *Intensive with access to outdoor run*  **Extensive** | 24 behavioural indicators related to Domains 1,2,4 grouped in functional systems: Social behaviour, locomotion, rest and sleep, feeding, elimination, comfort behaviour and exploration.  No comment on consistency or reliability of indicators but assessment is based on farmer report of housing system. | NACAH can be implemented based on farmer report without farm visit but farm classification for pig behaviour is based on pig housing systems present in Germany. |
| Sow Welfare (SOWEL) | Bracke et al, 2002  (Bracke et al. 2002) | The Netherlands | Describes the development of the SOWEL model to assess overall welfare of pregnant sows and desktop application to the seven main sow housing systems. SOWEL is a computer-based decision support system that takes a description of a housing and management system based on observation on farm as input and produces a welfare score as output. | Pregnant sows  Intensive indoors in thermocontrolled building (individual and group)  **Outdoor huts**  Family pen groups in open-fronted building | 37 attributes that assessed Domains 1-4 at the housing system level based on properties of housing and management.  No comment on consistency or reliability. | Involves on farm observation and farmer report of housing and management and data entry into SOWEL software, with levels per attribute based on pig housing systems present in The Netherlands. |
| Bien-Être en Élevage de Porcs (BEEP) – translates to Welfare in Pig Farming | Courboulay et al, 2020  (Courboulay et al. 2020) | France | Co-design of a pig welfare assessment tool for farmers within a collaborative project involving farmers, technical advisers and animal welfare science experts, and pilot on 14 farms. Purpose to raise farmer awareness of animal welfare issues and to provide a suitable protocol to help them improve the wellbeing of their own livestock. | Growing pigs  Intensive^b^  *Organic^c^* | 12 indicators grouped by WQ protocol principle that assessed Domains 1-4 involving group assessments and individual assessments.  No evaluation of tool consistency or reliability. | Easy to use by farmers after short training with a maximum duration of two hours for on-farm assessment. The indicators are consistent with the observations made by farmers during regular care of their animals.  Tool is designed for confined housing and management systems. |
| ‘Real Welfare’ Scheme | Pandolfi et al, 2017  (Pandolfi et al. 2017) | United Kingdom | ‘Real Welfare’ protocol to assess pig welfare on finishing farms using animal-based measures. The paper reports the results from the first 3 years of application in the Red Tractor Assurance Scheme. | Finishing pigs  Intensive^b^  *In&Outdoor (trobridge or kennel + yard)*  **Outdoor (shelter + field)** | Five animal-based measures that assessed Domains 3-4 recorded at pen-level.  Good inter-observer reliability assumed but not measured. | The protocol involves observation of groups of pigs during a relatively short on farm visit.  Somewhat limited in scope as it does not consider resource- or management-based measures. |
| Herd Health and Welfare Index (HHWI) | Wadepohl et al, 2019  (Wadepohl et al. 2019) | Germany  Netherlands  Belgium  Bulgaria  Denmark  France  Italy  Poland  Switzerland | Development and first results of the HHWI used to assess the quality of health and welfare of pig herds in nine European countries. Indicator selection considered length of assessment time and feasibility for routine use at herd level. | Farrow-to-Finisher  Intensive^b^ | Five animal-based measures (bursa alterations, lameness, manure on body, runts, tail/ear/flank biting) that assessed Domains 1&3 for two categories of pigs on farm to ascertain overall HHWI points per farm.  Pilot demonstrated reasonable inter-observer reliability within country and virtually no missing data. | Simplified assessment using only animal-based on-farm measures proven to be a feasible and sufficient for indicating  animal health and welfare differences between farms within a country with total assessment time of approximately 2 hours.  by trained assessor.  However, tool is designed for intensive confined housing and management systems. |
| Model Code of Practice for the Welfare of Animals Pigs Third Edition PISC Report No 92 Australia | Primary Industries Standing Committee, 2008  (Primary Industries Standing Committee 2008) | Australia | Australian Model Code of Practice for the Welfare of Animals prepared by the Animal Welfare Working Group (AWWG) within the Primary Industries Ministerial Council (PIMC) designated Standards as a guide for all people responsible for the welfare of pigs under intensive, deep litter and outdoor systems.  Detailed Standards that form the basis for assessment of compliance with good welfare and may be used by qualified auditors and inspectors to examine and judge pig welfare. Noting that the additional sections in addition to the Standards on Recommended practice and Guidelines are advisory only. | All pigs  Intensive^b^  Intensive deep litter  **Extensive** | Standards cover Domains 1-4 with sections for food and water, accommodation, equipment, environment, protection, waste control, pigs kept outdoors, inspections, health, farrowing and weaning, moving pigs, elective husbandry procedures, preparation for transport and slaughter, emergency euthanasia. | Use of standards for welfare assessment involves on farm visit and discussion with farmer. Standards are written based on pig housing systems present in Australia. |
| Scientific opinion on the use of animal-based measures to assess welfare in pigs | European Food Safety Authority (EFSA) Panel on Animal Health and Welfare 2012  (EFSA Panel on Animal Health and Welfare 2012) | European Union | European Food Safety Authority provides scientific opinion on the use of animal-based and non-animal-based measures to assess welfare in pigs through the Panel on Animal Health and Welfare.  Provides a comprehensive list of animal-based measures as a ‘toolbox’ from which the most appropriate measures for the objectives of an assessment of pig welfare can be selected. Recommendations on approach to implementation are presented such as:  Assessment using animal-based measures and non-animal-based measures where the non-animal measures are strongly associated with welfare outcome and are more efficient to measure.  A short list of measures for the initial welfare assessment in the first stage of a program is stated for each pig category.  Systematic approach to pig welfare assessment  Training of assessors to ensure valid and reliable measurement. | Short list of measures for  Fattening pigs  Sows and boars  Piglets  Housing system not specified | The short list of measures for the initial welfare assessment directly covers:  Domains 2-4 for fattening pigs (disease signs, skin lesions, tail and ear lesions, exploratory behaviour, the group of measures related to thermoregulation)  Domains 1-4 for sows and boars (skin lesions, body condition, persistent investigatory behaviour and stereotypies, locomotion score, disease signs)  Domains 1-3 for piglets (mortality, the group of measures related to thermoregulation, disease signs, the group of measures related to mutilation). | Initial welfare assessment using the short list of measures involves on farm visit and discussion with farmer.  EFSA scientific opinion and recommendations are written based on pig farming and housing systems present in countries of the European Union. |
| Council Directive 2008/120/EC | Council Directive 2008/120/EC of 18 December 2008 laying down minimum standards for the protection of pigs  (Council of the European Union 2009) | European Union | Animal welfare is a priority for the European Union and the welfare of pigs is assured by this Council Directive that applies to all categories of pigs and lays down the minimum standards for their protection.  The competent authority of each Member State is to conduct inspections on a regular basis to check that the provisions of this Directive are being complied with. | All pigs  Conventional farming systems, emphasis on indoors | The Directive covers Domains 1-4 with particular emphasis on Domain 2 housing design impacts on welfare and health of the animals. It includes Articles referring to Housing, Stockmanship, Hygiene, Food safety, Behaviour, Feed & water, Procedures, Provisions for specific pig categories. | Competent authority welfare inspection using a code based on this Directive involves on farm visit and discussion with farmer. Directive Articles are written based on pig housing systems present in countries of the European Union. |
| Code of Practice for the Welfare of Pigs | Department for Environment Food and Rural Affairs 2020  (Department for Environment Food and Rural Affairs 2020) | England | Code of Practice which applies to England only covering all farmed pigs. The Code helps owners/ keepers of pigs comply with animal welfare legislation.  Adherence to the Code recommendations will help keepers to maintain the standards required to comply with relevant legislation.  During on-farm welfare inspections carried out by the Animal and Plant Health Agency and Local Authorities, inspectors assess compliance against legislation and this Code. | All farmed pigs  Conventional farming systems  Intensive^b^ **Extensive** | Standards cover Domains 1-4 with detailed sections for Stockmanship & staffing, Health & welfare, Disease control & biosecurity, Emergencies, Inspection, Handling, Tethering, Transport, Marking, Responsible medicines usage & record keeping, Accommodation, Management, Mutilations, Additional specific recommendations per pig category, Outdoor husbandry systems. | Use of standards for welfare assessment involves on farm visit and discussion with farmer. Standards are written based on pig housing systems present in England. |
| Terrestrial Animal Health Code – Animal Welfare and Pig Production Systems | World Organisation for Animal Health (OIE) Terrestrial Animal Health Code 2019  (World Organisation for Animal Health 2019) | International | The OIE is the intergovernmental organisation responsible for improving animal health worldwide. The Terrestrial Animal Health Code provides standards for the improvement of animal health, animal welfare and veterinary public health to be applied by OIE Member Countries to safeguard the health and welfare of terrestrial animals and ensure the safety of international trade in animals and animal products.  Chapter 7.13 of the Code addresses the welfare aspects of commercial domestic pig production systems, with Article 7.13.4 specifying criteria for use to monitor the welfare of pigs. | Commercial domestic pig production systems  Intensive^b^ **Extensive**  *Indoor/outdoor combination systems* | Article 7.13.4 of the Code specifies 9 animal-based criteria that are useful indicators of animal welfare and cover Domains 1-4. | Assessment of the 9 criteria will involve on farm visit and farm records, plus require a considerable time allocation to complete.  Criteria are based on commercial domestic pig production systems. |

Alpigiani I, Bacci C, Keeling LJ, Salman MD, Brindani F, Pongolini S, Hitchens PL,Bonardi S, (2016) The associations between animal-based welfare measures and the presence of indicators of food safety in finishing pigs. Animal Welfare 25:355-363

Andronie I, Parvu M, Andronie V,Parvu V, (2014) Assessment of animal welfare - starting point for sustained improvement of their quality of life. Scientific Papers: Animal Science and Biotechnologies 47:156-159

Annen DN, Wieck C,Kempen M, (2011) Evaluation of minimum animal welfare conditions in national standards and farm certification schemes for pig fattening. Acta Agricultura Scandinavica. Section A, Animal Science 61:40-54

Barbari M, Gastaldo A,Rossi P, (2008) Farm welfare index for assessment of wellbeing in swine farms. (Ragusa Safety Health Welfare, Ragusa, Italy)

Ben-Dov D, Hadani Y, Ben-Simchon A, Alborali L,Pozzi PS, (2014) Guidelines for pig welfare in Israel. Israel Journal of Veterinary Medicine 69:4-15

Bergschmidt A,Schrader L, (2009) Application of an animal welfare assessment system for policy evaluation: Does the Farm Investment Scheme improve animal welfare in subsidised new stables? Landbauforschung Volkenrode 59:95-103

Bracke MBM, Spruijt BM, Metz JHM,Scheuten WGP, (2002) Decision support system for overall welfare assessment in pregnant sows A: Model structure and weighting procedure. Journal of Animal Science 80:1819-1834

Council of the European Union, (2009) Council Directive 2008/120/EC of 18 December 2008 laying down minimum standards for the protection of pigs (Codified version). 2009, 5-13

Courboulay V, Meunier-Salaün MC, Stankowiak M,Pol F, (2020) BEEP: An advisory pig welfare assessment tool developed by farmers for farmers. Livestock Science 240:104107-104114

Czycholl I, Grosse Beilage E, Henning C,Krieter J, (2017) Reliability of the qualitative behavior assessment as included in the Welfare Quality® assessment protocol for growing pigs. Journal of Animal Science 95:3445-3454

Czycholl I, Kniese C, Büttner K, Beilage E, Schrader L,Krieter J, (2016) Interobserver reliability of the ‘Welfare Quality® Animal Welfare Assessment Protocol for Growing Pigs’. SpringerPlus 5:1-13

Czycholl I, Kniese C, Büttner K, Grosse Beilage E, Schrader L,Krieter J, (2016a) Test-retest reliability of the Welfare Quality® animal welfare assessment protocol for Growing Pigs. Animal Welfare 25:447-459

Czycholl I, Kniese C, Schrader L,Krieter J, (2017a) Assessment of the multi-criteria evaluation system of the Welfare Quality® protocol for growing pigs. Animal 11:1573-1580

Czycholl I, Kniese C, Schrader L,Krieter J, (2018) How reliable is the multi-criteria evaluation system of the Welfare Quality® protocol for growing pigs? Animal Welfare 27:147-156

Department for Environment Food and Rural Affairs, (2020) Code of practice for the welfare of Pigs. 2020, England), 1-59

Dippel S, Leeb C, Bochicchio D, Bonde M, Dietze K, Gunnarsson S, Lindgren K, Sundrum A, Wiberg S, Winckler C,Prunier A, (2014) Health and welfare of organic pigs in Europe assessed with animal-based parameters. Organic Agriculture 4:149-161

EFSA Panel on Animal Health and Welfare, (2012) Scientific Opinion on the use of animal‐based measures to assess welfare in pigs. European Food Safety Authority Journal 10:2512

Friedrich L, Krieter J, Kemper N,Czycholl I, (2019) Test−retest reliability of the ‘Welfare Quality® animal welfare assessment protocol for sows and piglets’. Part 1. assessment of the welfare principle of ‘appropriate behavior’. Animals 9

Friedrich L, Krieter J, Kemper N,Czycholl I, (2019a) Test-retest reliability of the Welfare Quality® Assessment protocol for pigs applied to sows and piglets. Part 2. Assessment of the principles good feeding, good housing, and good health. Journal of Animal Science 97:1143-1157

Friedrich L, Krieter J, Kemper N,Czycholl I, (2020) Iceberg indicators for sow and piglet welfare. Sustainability 12:1-24

Friedrich L, Krieter J, Kemper N,Czycholl I, (2020a) Animal welfare assessment in sows and piglets— introduction of a new german protocol for farm’s self‐inspection and of new animal‐based indicators for piglets. Agriculture 10:1-14

Losada-Espinosa N, Trujillo-Ortega ME,Galindo F, (2017) The welfare of pigs in rustic and technified production systems using the welfare quality protocols of pigs in mexico: Validity of indicators of animal welfare as part of the sustainability criteria of pig production systems. Veterinaria Mexico 4

Martin P, Czycholl I, Buxade C,Krieter J, (2017) Validation of a multi-criteria evaluation model for animal welfare. Animal : an international journal of animal bioscience 11:650-660

Munsterhjelm C, Heinonen M,Valros A, (2015) Application of the Welfare Quality® animal welfare assessment system in Finnish pig production, part I: Identification of principal components. Animal Welfare 24:151-160

Pandolfi F, Kyriazakis I, Stoddart K, Wainwright N,Edwards SA, (2017) The “Real Welfare” scheme: Identification of risk and protective factors for welfare outcomes in commercial pig farms in the UK. Preventive Veterinary Medicine 146:34-43

Pfeifer M, Koch A, Lensches C, Schmitt AO,Hessel EF, (2020) Acceptance and feasibility of a guideline for the animal welfare assessment of fattening pigs from farmers’ point of view. Animals 10

Primary Industries Standing Committee, (2008) Model Code of Practice for the Welfare of Animals Pigs. 2008, Collingwood, Victoria),

Scott K, Binnendijk GP, Edwards SA, Guy JH, Kiezebrink MC,Vermeer HM, (2009) Preliminary evaluation of a prototype welfare monitoring system for sows and piglets (Welfare Quality project). Animal Welfare 18:441-449

Temple D, Courboulay V, Velarde A, Dalmau A,Manteca X, (2012) The welfare of growing pigs in five different production systems in France and Spain: assessment of health. Animal Welfare 21:257-271

Temple D, Dalmau A, Ruiz de la Torre JL, Manteca X,Velarde A, (2011) Application of the Welfare Quality® protocol to assess growing pigs kept under intensive conditions in Spain. Journal of Veterinary Behavior: Clinical Applications and Research 6:138-149

Temple D, Manteca X, Dalmau A,Velarde A, (2013) Assessment of test-retest reliability of animal-based measures on growing pig farms. Livest Sci 151:35-45

van Staaveren N, Calderón Díaz JA, Garcia Manzanilla E, Hanlon A,Boyle LA, (2018) Prevalence of welfare outcomes in the weaner and finisher stages of the production cycle on 31 Irish pig farms. Irish Veterinary Journal 71

Wadepohl K, Blaha T, Van Gompel L, Duarte ASR, Nielsen CL, Saatkamp H, Wagenaar JA,Meemken D, (2019) Development of a simplified on-farm animal health and welfare benchmarking tool for pig herds. Berliner und Munchener Tierarztliche Wochenschrift 132:504-512

World Organisation for Animal Health, (2019) Chapter 7.13. Animal Welfare and Pig Production Systems. Terrestrial Animal Health Code, 2019, (OIE, pp 1-14
